# Response to a Comment by Albert et al. (*Nutrients* 2017, *9*, 137) Entitled “Concerns with the Study on Australian and New Zealand Fish Oil Products” by Nichols et al. (*Nutrients* 2016, *8*, 703)

**DOI:** 10.3390/nu9060583

**Published:** 2017-06-07

**Authors:** Peter D. Nichols, Lalen Dogan, Andrew J. Sinclair

**Affiliations:** 1 CSIRO Oceans and Atmosphere, GPO Box 1538, Hobart, TAS 7000, Australia; 2 DSM Nutritional Products Asia Pacific, 30 Pasir Panjang Road, Mapletree Business City, #13-31, Singapore 117440, Singapore; lalen.dogan@dsm.com; 3 School of Medicine, Deakin University, Geelong, VIC 3220, Australia; andrew.sinclair@deakin.edu.au; 4 Department of Nutrition, Dietetics & Food, Monash University, Clayton, VIC 3800, Australia

The Comment by Albert et al., 2017 [1] raises five issues on our 2016 *Nutrients* paper [2]. The Nichols et al., 2016 paper [2] was prepared following publication of Albert et al. [3] in *Scientific Reports* by the Liggins Institute titled “Fish oil supplements in New Zealand are highly oxidised and do not meet label content of *n*-3 PUFA”. We respond to the five issues raised in [1] as follows. 

“First, the 10 products studied represent only a fraction of more than 40 fish oil products available in Australasia, so that the study led by the Omega-3 Centre (O3C) covered substantially fewer products than in other recent publications. This unexplained small sample size (even if chosen based on market share) suggests a possible selection bias, making the survey unrepresentative of the range of products available to consumers.” 

*Response 1*: We reject the suggestion of sample bias. The 10 products selected for analysis by the O3C represented over 80% of the Australian market, which overlaps considerably with the New Zealand market. The larger Bannenberg et al., 2017 study of >40 New Zealand samples [4], which we were aware of at the time and was cited in [2], showed results in full agreement with Nichols et al., 2016 [2], as does one further 2017 New Zealand study of 11 products [5] and a previous study of 15 products by Australia’s Therapeutic Goods Administration (TGA) [2]. 

“Second, the methodology used in the measurement of fatty acid concentration is insufficiently described for the study to be replicated. The authors stated in their article that “full details on analytical methods are available on request from ALS” [1], which was the company subcontracted to perform the analyses (ALS Food and Pharmaceutical). ALS have provided their method on condition of confidentiality, which limits the detail we can provide of our assessment of their method. Nevertheless, we find that it lacks description of key features necessary to determine fatty acid concentration in mg/g of oil, which is required in order to determine actual EPA and DHA content. If a quantitative method of fatty acid analysis was not used, these data should be retracted. Further, we note that the method of fatty acid analysis remains unavailable to the scientific community.” 

*Response 2*: We reject the statement that the oils were incorrectly analysed. The TGA requires oils and omega-3 to be analysed using standard methods. ALS is a certified laboratory, and uses methods approved by TGA [2]. The TGA certified ALS Methods were submitted as supplementary material to *Nutrients* in the review process and indicated the use of internal standards, as did the Nichols et al. Nutrients paper [2]; therefore—fatty acid concentrations in mg/g could be calculated. Albert et al. [3] did not use certified methods. ALS had sent the full methods to Albert et al. [3], so the statement in the Comment is not correct. The data in [2] are not retracted. 

“Third, Nichols et al. incorrectly characterise the methodology used in our own study [3] as non-standard. In our study, peroxide and anisidine values were determined in strict accordance with the methods of the European Pharmacopoeia. Further, fatty acid content was determined using the quantitative method first described by Lepage and Roy [6]. This method is highly cited in the peer-reviewed literature (nearly 1600 Scopus citations) and widely used in studies involving fatty acid analysis of food fats, supplements, and biological fluids/tissues, published in some of the world’s top scientific journals (such as *New England Journal of Medicine, JAMA, Cell Metabolism, Proceedings of the National Academy of Sciences of the USA*, and the *American Journal of Clinical Nutrition*).” 

*Response 3*: The methods described in [3] are not TGA/industry accepted methods. We recommend that Albert et al. contact the TGA to clarify this point. Laboratory proficiency programs are available as are appropriate standards; we also recommend that Albert et al. validate their methods for oils and ethyl esters. Further, Bannenberg et al. [4] have raised other methodological concerns with the Albert et al., 2015 study [3], including possible issues with their FA method [6] and sample preparation. 

“Fourth, Nichols et al. have supported their findings by citing personal communications and unpublished data, but they appear to have overlooked all of the independent studies from around the world showing under-delivery of *n*-3 PUFA content and high levels of oxidation in retail fish oil products. These studies indicate that 17–93% of fish oil products exceed recommended limits of primary oxidation at the time of purchase. It is also important to clarify that the Ismail et al. review [7] does not include “over 2000 published analyses” showing low levels of oxidation in fish oil products as stated by Nichols et al. [2]; rather, the review in question only cites a figure based on unpublished data that have not been peer-reviewed. Furthermore, it is interesting that Nichols et al. observed a high rate of secondary oxidation in their own study (even after excluding flavoured products): the proportion of products exceeding the Global Organization for EPA and DHA Omega-3s (GOED) limit for anisidine value (3/8 or 37.5%) [2] was greater than in our study [3] and similar to a North American investigation. In regards to labelled content, numerous studies have shown that under-delivery of n-3 PUFA content (i.e., <90% labelled) is common worldwide.” 

*Response 4*: We reject the statement that selected reporting of the literature occurred in Nichols et al. [2]. The GOED study on New Zealand products referred to in [2] was published by Bannenberg et al., 2017 [4], with >40 samples analysed, and the findings are in full agreement with Nichols et al., 2016 [2]. Bannenberg et al. [4] raised concerns, including inadequate description of methods and lack of validation for ethyl ester and oil samples, about the Albert et al. paper [3]. A detailed comparison of results in Albert et al. [3] has been performed by Bannenberg et al. [4] and clearly shows that the majority of products are not oxidized and generally meet their omega-3 label content claims, in full agreement with our study [2]. 

“Lastly, it is baffling how the authors could have declared no conflicts of interest, when this is quite clearly not the case. Both Nichols and Sinclair are scientific advisors to the O3C, while Dogan is the Chair of the O3C and also a director (Nuritional Lipids, Asia Pacific) of DSM Nutritional Products. The latter is a multinational corporation that sells nutritional supplements (including algal and fish oil), while the stated mission of the O3C includes “supporting the development of the market for (…) dietary supplements containing long chain omega-3s”. Failure to declare these conflicts breaches international standards of transparency in publishing. There is concern about conflict of interest and how it effects the scientific literature, with evidence that studies influenced by industry are biased towards showing favourable results.” 

*Response 5*: O3C is a not for profit organization, including with an unpaid board and scientific advisors, the latter from academia. The board is elected by O3C members at the annual general meeting. The O3C operates as a centre of excellence in omega-3 fatty acids for Australia and New Zealand and represents the broad interests of researchers and companies in this field. The primary focus is on communicating the health benefits of omega-3. Full details of the O3C are available at [8]. 

In summary, the statements in the Comment by Albert et al., 2017 [1] on our paper (Nichols et al., 2016) [2] are selective, inaccurate and incorrect. As has been demonstrated by multiple studies, in marked contrast to Albert et al., 2015 [3], the majority of Australian and New Zealand fish oil products **do meet** their omega-3 label claims and **are not oxidized** as reported in four separate studies ([2,4,5] TGA: 2).
